# Knowledge, Attitudes and Practices of Health Workers and Caregivers Towards Retinopathy of Prematurity in Uganda: A Mixed‐Methods Study

**DOI:** 10.1002/puh2.208

**Published:** 2024-07-02

**Authors:** Rebecca Claire Lusobya, Immaculate Atukunda, Andrew Weil Semulimi, Carol Nalukenge, Abubaker Kalinaki, Erima Denis, Mary Nyanzi, Charles Batte, David Mukunya, John Mukisa, Juliet Otiti‐Sengeri, Geoffrey Wabulembo

**Affiliations:** ^1^ Department of Ophthalmology School of Medicine College of Health Sciences Makerere University Kampala Uganda; ^2^ Lung Institute Department of Medicine School of Medicine College of Health Sciences Makerere University Kampala Uganda; ^3^ Department of Ophthalmology Uganda Martyrs’ Hospital Lubaga Kampala Uganda; ^4^ Department of Paediatrics Kawempe National Referral Hospital Kampala Uganda; ^5^ Department of Community and Public Health Faculty of Health Sciences Busitema University Mbale Uganda; ^6^ Department of Immunology and Molecular Biology School of Biomedical Sciences College of Health Sciences Makerere University Kampala Uganda; ^7^ Light for the World International Kampala Uganda

## Abstract

**Introduction:**

Retinopathy of prematurity (ROP) is a significant global issue and a leading cause of preventable childhood blindness. Early screening and timely management of preterm babies at risk are crucial. To effectively implement this strategy, it is essential that caregivers and health workers are well‐informed about ROP. This study plays a vital role in assessing the knowledge, attitudes and practices of caregivers and healthcare workers towards ROP in Uganda, providing valuable insights into the current understanding and approach towards this condition.

**Method:**

We conducted a sequential explanatory mixed‐methods study, involving 214 participants. This group included 12 paediatricians, 56 neonatal nurses and 146 caregivers, all of whom play crucial roles in the healthcare system. The study was conducted at two tertiary hospital neonatal clinics in Uganda. A researcher‐administered structured questionnaire was used to collect the data on the participants’ knowledge, attitudes and practices. Descriptive statistics were used to describe variables, while qualitative data were analysed using thematic content analysis.

**Results:**

We recruited 146 caregivers with a mean age of 29.4 (±standard deviation 6.5) years, 12 paediatricians with a median age of 37 years (IQR: 36–41) and 56 nurses with a median age of 35 years (30–42). The median working duration of health workers at the neonatal unit was 3 (IQR: 1.8–4.2) years. Thirty‐two (21.92%) caregivers and 28 (49.12%) health workers had good knowledge about ROP, whereas 9 nurses and 2 paediatricians did not know about ROP. Barriers to ROP screening included limited resources (equipment, time and skilled personnel) and limited parental involvement. Enhancing collaboration among medical care teams has the potential to improve ROP screening.

**Conclusion:**

Insufficient knowledge about ROP among caregivers calls for increased efforts to educate and sensitise them about ROP and its risk factors.

## Graphical abstract

• Caregivers, paediatricians and neonatal nurses had insufficient knowledge about retinopathy of prematurity (ROP) and most had training about it.

• Minimal sensitisation of the caregivers and health workers about ROP and heavy workload experienced by the paediatricians and neonatal nurses were barriers towards prevention of ROP.

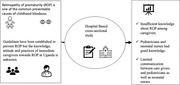

## 1 Introduction

Retinopathy of prematurity (ROP) is the leading cause of preventable childhood blindness in premature infants [1, 2, 3]. In 2010, global ROP diagnoses exceeded 184,700, resulting in 50,000 cases of childhood blindness [4]. Countries in the sub‐Saharan region, such as Kenya (41.7%) and Botswana (11%), have a high prevalence of ROP [5, 6, 7]. Despite the significant burden, only two African countries, Kenya and South Africa, have national ROP screening and prevention policies [8].

Early identification enhances at‐risk infants’ visual functioning and structural outcomes [9]. The WHO advises that trained personnel should perform a fundus examination for high‐risk infants within 6–7 weeks of delivery [1]. However, challenges persist in implementing this recommendation, as only 40% of neonatal intensive care units in LMIC settings have established screening programmes [4, 8]. Advocating for enhanced knowledge, attitudes and practices is crucial to implementing this strategy and achieving VISION beyond 2020 goals, particularly in Uganda [9]. This study evaluated caregivers’ and healthcare workers’ knowledge, attitudes and practices concerning ROP in Uganda.

## 2 Methods

### 2.1 Study Design

We conducted a multicentre cross‐sectional study between March and May 2022. This was later followed by qualitative data collection to understand and explore the quantitative results.

### 2.2 Study Setting

The study was conducted in two neonatal clinics at Kawempe National Referral Hospital (KNRH) and Mulago Specialised Women and Neonatal Hospital (MSWNH) in Kampala, Uganda. The two facilities are close to the Mulago National Referral Hospital eye clinic, which can provide eye examination services to preterm infants. The MSWNH clinic is run once a week by 9 paediatricians with 25 neonatal nurses, whereas the KNRH clinic is run daily by 6 paediatricians with 29 neonatal nurses. The KNRH clinic reviews approximately 100 preterm babies weekly, and the MSWNH clinic examines a maximum of 20 weekly. In both hospitals, the preterm babies are reviewed every 2 weeks and are discharged after attaining 2.5 kg. Both hospitals receive a relatively high number of preterm infants from all parts of the country but have no ophthalmologist or protocols for ROP screening and management.

### 2.3 Study Population and Eligibility Criteria

All paediatricians, neonatal nurses (MSWNH and KNRH) and caregivers of preterm infants attending the neonatal clinics at the two hospitals during the study period who met the eligibility criteria were enrolled. We selected paediatricians and nurses from the neonatal clinic who consented to the study. Caregivers of preterm infants at risk for ROP (born before 37 weeks gestational age or less than 2 kg birth weight) attending the neonatal clinics during the study period and consenting to participate were included. Exclusions were made for those unable to complete the full interview.

### 2.4 Sample Size Estimation

After calculating the sample size for proportions [10], 214 consecutive participants were enrolled in this study. Twelve paediatricians, 56 nurses and 146 caregivers of preterm infants at risk for ROP were enrolled. There was no formal sample size calculation for the paediatricians and neonatal nurses involved in the in‐depth interview. We enrolled all 12 paediatricians and 56 neonatal nurses at the two hospitals.

### 2.5 Study Variables

#### 2.5.1 For Paediatricians and Nurses

The study collected participant data on social demographics, including age, sex, designation and duration of practice in the neonatal unit. Knowledge assessment involved determining their understanding of ROP, its risk factors, preventive measures and methods for screening and treatment. Attitude evaluation was focused on their responses regarding the necessity of eye examination for preterm infants, who should perform fundus examinations and the need to inform caregivers about ROP. Practice patterns were assessed based on their answers regarding the number of premature babies examined, at‐risk babies referred to an ophthalmologist and the number of attempted fundus examinations.

#### 2.5.2 For Caregivers

Collected variables included social demographics such as age, sex, education level, baby age and birth weight. Caregiver knowledge was assessed through their responses to questions regarding ROP description, prevention, risk factors, screening and treatment.

### 2.6 Study Procedures

Data collection involved using an investigator‐administered semi‐structured questionnaire for paediatricians and nurses developed based on previously published literature [11, 12, 13]. Basic knowledge of ROP is essential for the timely referral of preterm infants for examination, which is a responsibility shared by both groups. Additionally, nurses play a crucial role in health education and counselling in Uganda. Qualitative data regarding the health workers’ barriers and challenges to ROP screening were collected during in‐depth interviews using an investigator‐administered questionnaire based on previously published literature [11, 14]. All health workers participated in the qualitative data collection and the in‐depth interviews. The interviews were led by a moderator (DK), assisted by a note taker (CN), each lasting 30 min.

Data for the caregivers were collected using a different questionnaire based on published literature [15]. Primary caregivers were not included in the in‐depth interviews. Trained research assistants collected all data.

### 2.7 Data Analysis

#### 2.7.1 Quantitative Data Analysis

Descriptive statistics were calculated for continuous variables using medians and interquartile ranges, while proportions were done for categorical data. Separate analyses were done for the caregivers and healthcare workers. The caregivers’ and healthcare workers’ knowledge of ROP was summarised as proportions. Knowledge about ROP was evaluated based on their selection of appropriate options. For instance, in defining ROP, paediatricians and neonatal nurses who selected all three correct options were categorised as having good knowledge. At the same time, those who chose two were deemed to have fair knowledge, and those who selected one or none were classified as having poor knowledge. This same method was applied to assess the knowledge of the two categories of participants regarding the risk factors for ROP. Similarly, for factors contributing to ROP prevention, participants were grouped based on their selection of options. Those who chose more than five options were considered to have good knowledge, three to four options indicated fair knowledge and less than three options reflected poor knowledge. Regarding treatment, good knowledge was determined by selecting at least one option, whereas poor knowledge was indicated by selecting none.

#### 2.7.2 Qualitative Data Analysis

Qualitative data were analysed using Open Code software to determine the barriers and opportunities to care for ROP among healthcare workers. Audio recordings were transcribed verbatim from Luganda to English by a social anthropologist competent in both Luganda and English. The principal investigator examined the recordings and transcripts to ensure that they were consistent with the audio files. The transcripts were repeatedly read through to ensure that they were correct and the responses were complete. The qualitative data were analysed using the content thematic approach in line with the study objectives. Transcripts were read several times to identify themes and sub‐themes. Coding was done by grouping data in line with the themes and sub‐themes and presenting selected voices/quotes.

### 2.8 Ethics Consideration

Ethics approval was sought from the Mulago Hospital Research and Ethics Committee (MHREC‐2228) and the Uganda National Council of Science and Technology (HS2228ES) before data collection and administrative clearance were granted by the tertiary hospitals. Written informed consent was obtained before the initiation of study procedures, which followed the principles of the Declaration of Helsinki and Ugandan laws and regulations. The study team members were trained in Good Clinical Practice principles and Human Subject Protection.

## 3 Results

### 3.1 Socio‐Demographic Characteristics of the Study Participants

#### 3.1.1 Caregivers and Preterm Infants

A total of 146 caregivers with a mean age of 29.4 ± 6.5 years were enrolled in the study. Table 1 summarises participant characteristics. The mean gestational age at birth of the babies included in this study was 30.4 ± 3 weeks, whereas the median age of the babies at the time of recruitment into the study was 6.6 (4–13.1) weeks.

**TABLE 1:** Socio‐demographics characteristics of participants who participated in the study at two neonatal clinics in Kampala, Uganda, March–May 2022.

Characteristics	Frequency (%)	Mean ± SD	Median, interquartile range
Caregivers			
Age of caregivers (years)		29.4 ± 6.5	
Sex of caregivers			
Female	144 (98.6)		
Male	2 (1.4)		
Relationship with the baby			
Mother	142 (97.2)		
Father	2 (1.4)		
Other	2 (1.4)		
Marital status			
Married	127 (87.0)		
Separated/divorced	1 (0.7)		
Single mothers/fathers	18 (12.3)		
Level of education, *n* = 145*			
None	3 (2.1)		
Primary	30 (20.5)		
Secondary	60 (41.1)		
Tertiary	52 (35.6)		
Missing	1 (0.7)		
Residence			
Rural	9 (6.2)		
Urban	137 (93.8)		
Employment status			
Unemployed	73 (50.0)		
Employed	73 (50.0)		
Gestational age of the baby at birth (weeks)		30.4 ± 3	
Age of the baby at the inclusion into the study (weeks)			6.6 (4–13.1)
Sex of the baby			
Female	82 (56.2)		
Male	64 (43.8)		
Paediatricians and neonatal nurses			
Age of paediatricians (years)			37 (36–41)
Age of nurses age (years)			35 (30–42)
Sex			
Female	58 (85.29)		
Male	10 (14.71)		
Position			
Paediatrician	12 (17.65)		
Nurse	56 (82.3)		
Overall duration of practice at the neonatal unit			3 (1.8–4.2)
Duration of practice by nurses (years)			3 (1.8–4.7)
Duration of practice by paediatricians (years)			3 (1.9–4)

^*^ Only 145 of the 146 caregivers responded to this question.

#### 3.1.2 Knowledge About ROP Among Caregivers

Only 32 (21.9%) caregivers knew about ROP (Table 2). Of the 32 caregivers, three‐quarters (24) could define ROP excellently and knew that it was preventable. Just over half (17) who were knowledgeable about ROP had good knowledge about the risk factors of ROP.

**TABLE 2:** The knowledge of caregivers about retinopathy of prematurity (ROP) in this study, conducted at two neonatal clinics in Kampala, Uganda, from March to May 2022.

Characteristics	Frequency (%)
Do you know about ROP?	
Yes	32 (21.9)
Definition of ROP (*n* = 32)	
Excellent: immature growth of vessels at the back part of the eye (retina) due to prematurity	24 (75.0)
Good: immature growth of the back of the eye (retina) caused by prematurity	8 (25.0)
Risk factors of ROP in babies^a^	
Good	17 (53.1)
Fair	4 (12.5)
Poor	11 (34.4)
How is ROP identified?	
Good (examining the back segment of the eye)	6 (18.75)
Fair (examining the eye)	20 (62.5)
Poor (I don't know)	6 (18.75)
When should the first eye examination be performed?	
Good (4–6 weeks of age)	28 (87.5)
Poor (other)	4 (12.5)

^a^ Risk factors for ROP included: low gestation age <32 weeks, birth weight <1.5 kg, sick babies requiring oxygen therapy (poor: one correct option or none, fair: two correct options, good: three correct options).

#### 3.1.3 Knowledge About ROP Among Health Workers Involved in Neonatal Care

The results on knowledge, as shown in Table 3, indicate that 28 (49.12%) of the 57 paediatricians and neonatal nurses had a good definition of ROP, with the highest percentage seen among paediatricians. More than one‐ninth (52) recruited paediatricians and neonatal nurses knew that it was preventable, whereas 50 (87.72%) responded that ROP is treatable. All the paediatricians (10, 100%) reported inadequate awareness and knowledge about ROP among medical, paramedical and caregivers of patients.

**TABLE 3:** The knowledge of paediatricians and neonatal nurses about retinopathy of prematurity (ROP) in this study conducted at two neonatal clinics in Kampala, Uganda, from March to May 2022.

Characteristics	Total, *n* (%)	Paediatricians, *n* = 10, *n* (%)	Nurses, *n* = 47, *n* (%)
Definition of ROP^a^			
Good	28 (49.1)	6 (60.0)	22 (46.8)
Fair	21 (36.8)	2 (20.0)	19 (40.4)
Poor	8 (14.0)	2 (20.0)	6 (12.8)
Which babies are likely to develop ROP?^b^			
Good	26 (45.6)	6 (60.0)	20 (42.5)
Fair	15 (26.3)	2 (20.0)	13 (27.7)
Poor	16 (28.1)	2 (20.0)	14 (29.8)
Is ROP preventable?			
Yes	52 (91.2)	10 (100.0)	42 (89.4)
Prevention of ROP in the first 4 h after childbirth^c^			
Good	34 (59.6)	9 (90.0)	25 (53.2)
How is ROP identified?			
Good (examining the retina)	29 (50.9)	10 (100.0)	19 (40.4)
Timing of the first eye ROP screening			
4–6 weeks of age/depends on the gestational age	33 (57.9)	8 (80.0)	25 (53.2)
Is ROP treatable?			
Yes	50 (87.7)	9 (90.0)	41 (87.2)
If yes, what is the treatment for ROP?^d^ (*n* = 50)			
Good	32 (64.0)	2 (22.2)	30 (73.2)
In your opinion, is there enough awareness and knowledge of ROP among medical and paramedical staff and patient caregivers?			
Yes	5 (8.8)	0 (0.0)	5 (10.6)

^a^ Definition of ROP: An eye condition that affects preterm babies, causes blindness if not treated and causes the development of abnormal vessels in the retina (good: three options mentioned, fair: two options, poor: one or none of the alternatives mentioned).

^b^ Risk factors for ROP: similar to that mentioned in Table 2.

^c^ Factors that contribute to ROP prevention within the first 4 h of birth: management of oxygen levels (that is 30% oxygen mix or air, not pure oxygen), management of transport of the premature baby from the labour suite to the neonatal unit, maintaining the babies’ temperature between 36.5° and 37.5° centigrade, Supporting the baby's respiratory system, management of temperatures in the delivery room, Stabilise the baby in the delivery room before transfer to the neonatal special care unit (good: >5 options mentioned as true, fair: 3–4 options mentioned as true, poor: <3 options mentioned as true).

^d^ Treatment for ROP includes laser, eye injection and surgery.

Concerning the attitude of paediatricians and neonatal nurses towards ROP (Table 4), close to three‐quarters (42) of them strongly agreed that preterm babies requiring oxygen supplementation should have frequent eye checks. Most (89.3%) of the nurses disagreed that an eye examination is only needed in preterm infants when vision is affected or when they have an eye complaint. All paediatricians and nurses agreed that they should inform the caregivers about ROP.

**TABLE 4:** The attitude of paediatricians and neonatal nurses who participated in a study conducted at two neonatal clinics in Kampala, Uganda, towards screening and managing retinopathy of prematurity (ROP), March–May 2022.

Attitude, *n* = 57	Total, *n* = 57, *n* (%)	Paediatricians, *n* = 10, *n* (%)	Nurses, *n* = 47, *n* (%)
An eye examination is only required in preterm infants when vision is affected			
Strongly agree	1 (1.8)	0 (0.0)	1 (2.1)
Agree	3 (5.3)	0 (0.0)	3 (6.5)
Disagree	31 (54.4)	5 (50.0)	26 (55.3)
Strongly disagree	21 (36.8)	5 (50.0)	16 (34.0)
Missing	1 (1.8)	0	1 (2.1)
An eye examination is only required in preterm infants with an eye complaint			
Strongly agree	1 (1.8)	0 (0.0)	1 (2.1)
Agree	2 (3.5)	0 (0.0)	2 (4.3)
Disagree	39 (68.4)	7 (70.0)	32 (4.3)
Strongly disagree	14 (24.6)	3 (30.0)	11 (23.4)
Missing	1 (1.8)	0 (0.0)	1 (2.1)
Fundus examination should be done by ophthalmologists only			
Strongly agree	14 (24.6)	0 (0.0)	14 (29.8)
Agree	18 (31.6)	0 (0.0)	18 (38.3)
Neutral	4 (7.0)	2 (20.0)	2 (4.3)
Disagree	18 (31.6)	8 (0.0)	10 (21.3)
Strongly disagree	1 (1.8)	0 (0.0)	1 (2.1)
Missing	2 (3.5)	0 (0.0)	2 (4.3)
Fundus examination by non‐ophthalmologists could help detect ROP in preterm			
Strongly agree	4 (7.0)	2 (20.0)	2 (4.3)
Agree	34 (59.6)	8 (80.0)	26 (55.3)
Neutral	5 (8.8)	0 (0.0)	5 (10.6)
Disagree	11 (19.3)	0 (0.0)	11 (23.4)
Strongly disagree	2 (3.5)	0 (0.0)	2 (4.3)
Missing	1 (1.8)	0 (0.0)	1 (2.1)
Preterm infants on oxygen supplementation require more frequent eye check‐up			
Strongly agree	42 (73.7)	6 (60.0)	36 (76.6
Agree	14 (24.6)	4 (40.0)	10 (21.3)
Missing	1 (1.8)	0 (0.0)	1 (2.1)
Neonatal nurses should inform caregivers of preterm babies about ROP			
Strongly agree	46 (80.7)	5 (50.0)	41 (87.3)
Agree	10 (17.5)	5 (50.0)	5 (10.6)
Missing	1 (1.8)	0 (0.0)	1 (2.1)
Paediatricians should inform caregivers of preterm babies about ROP			
Strongly agree	43 (75.4)	6 (60.0)	37 (78.7)
Agree	13 (22.8)	4 (40.0)	9 (19.1)
Missing	1 (2.1)	0 (0.0)	1 (2.1)

Close to one‐sixth (34) of the paediatricians and neonatal nurses agreed that fundus examination conducted by non‐ophthalmologists could help detect ROP in preterm babies. Regarding the practices of paediatricians and neonatal nurses (Table 5), only three (5.26) had attempted a fundus examination. Only 56% of paediatricians and 46% of nurses reported consistently referring premature infants to an ophthalmologist for ROP screening.

**TABLE 5:** The practices of paediatricians and neonatal nurses who took part in a study conducted at two neonatal clinics in Kampala, Uganda, concerning the screening and management of retinopathy of prematurity (ROP) from March to May 2022.

Practices, *n* = 57	Total, *n* (%)	Paediatricians, *n* = 10, *n* (%)	Nurses, *n* = 47, *n* (%)
Do you refer premature infants to an ophthalmologist?			
Yes	46 (80.7)	9 (90.0)	37 (78.7)
If yes, how often? *n* = 46			
Sometimes	19 (41.3)	4 (44.4)	15 (40.5)
Often	5 (10.9)	0 (0.0)	5 (13.5)
Always	22 (47.8)	5 (55.6)	17 (46.0)
Have you received any training about ROP prevention and screening?			
Yes	14 (24.6)	8 (80.0)	6 (12.8)
Do you counsel any caregivers of at‐risk babies about ROP?			
Yes	46 (80.7)	9 (90.0)	37 (78.7)
How many babies are being screened for ROP at your facility per week?			
None	23 (40.3)	5 (50.0)	18 (38.3)
1–5 babies per week	25 (43.9)	4 (40.0)	21 (44.7)
6–10 babies per week	6 (10.5)	0 (0.0)	6 (12.8)
Above 10 babies per week	3 (5.3)	1 (10.0)	2 (4.3)

### 3.2 Barriers and Opportunities to ROP Screening and Care

#### 3.2.1 Barriers

##### 3.2.1.1 Limited Parental Involvement in ROP Communication

According to the participants, there was minimal caregiver involvement in the ROP screening and care cascade. Some of them stated:

The neonatal team in our facility does not involve parents in the care of their babies to manage and prevent ROP.*—*Paediatrician _009

They do always involve them in the way of health educating them about the care of the eyes and some signs and symptoms that can show up that the mother can detect and take the baby to the hospital.—Nurse_015

##### 3.2.1.2 Lack of Equipment and Skills in ROP Screening

The participants highlighted that minimal ROP screening criteria were implemented at the NICU. They also noted that they have no equipment to screen for ROP, as illustrated in the following:

Because we do not have the machines to screen these babies, we provide health education to them and inform them that if you discover any situation that is not good with the baby's eyes, you should go to the hospital. We inform them on discharge that if they find out anything about the baby's eye or see as if the baby does not see well, please go to the ophthalmologist.—Nurse_018

##### 3.2.1.3 Lack of Adequate Time

The nurses and paediatricians cited the high number of preterm children at the NICU as a key barrier to providing ROP‐specific care. They were often too exhausted to screen for or refer the high‐risk children for ROP reviews and assessments. The following two quotes illustrate this:

The neonatal unit in KNRH has four cubicles; the first cubicle might have about 40 children on average. Moreover, there are two other cubicles: an Intensive Care Unit (ICU) where the ventilated babies are and a massive ventilator full of premature babies. The other is full of new preterm. There is so much action in the neonatal unit. And then the interaction that mothers should have been an interruption to our work so even when they do come to do the basic things like changing diapers, what you are thinking about at that point is not counselling about retinopathy or prematurity.—Nurse_008

We have not done an outstanding job because we often look out for complications that would take the baby's life at that point. So, when they come out of that, their breathing is stabilised, their heart is stabilised, and everyone is happy. So that is when we remember that we must do an eye exam. So many times, many babies would go without the eye exam.—Nurse_011

### 3.3 Opportunities

The nurses and paediatricians discussed possible opportunities for improving ROP screening at their hospitals. These included designing systems and care protocols for ROP screening and care, educating mothers about ROP, using a multidisciplinary approach to ROP and training health workers about ROP, especially in identifying at‐risk babies who need screening.

#### 3.3.1 Designing Systems and Care Protocols for ROP Care

The paediatricians noted that they had initiated protocols regarding oxygen delivery to preterm babies to protect them from ROP.

We are trying to optimise all the other interventions to reduce oxygen requirement in these children and therefore reduce the possibility of damage or reduce retinal damage from excessive oxygen or oxygen‐free radicals. We have protocols which we are improving. —Pediatrician_007

The nurses and paediatricians also reiterated that there are ongoing interventions to engage ophthalmologists to assess high‐risk children before discharge, as illustrated by the quotes in the following:

The eye doctor is called in to screen the babies who need screening before discharge. We make sure that we emphasise it to the parents to take them for screening.—Pediatrician_002

#### 3.3.2 Increase the Collaboration Among Paediatricians, Nurses, Ophthalmologists and Obstetricians

Competent teams improve the efficiency of ROP screening, referral and treatment. Some in‐depth participants noted a dire need for highly skilled individuals and professionals to identify and manage children at risk of developing ROP. Furthermore, a multidisciplinary approach is needed to combat this disease.

So, we are not doing what we could do, and that is why this kind of role should be with appropriate training extended beyond the paediatrician. There can only be so many paediatricians to do this. But, in terms of involving caretakers, if the nurses are well trained, they could give the right information, of course being careful not to implicate oxygen so much that people would rather not receive oxygen to save their eyes. Then, the child does not even live. So, it is a delicate balance, but it needs a multidisciplinary team because we have so many patients. – Pediatrician_003.

Many of my doctors do not know how to examine the eye. So, the fundoscopy eye examination is strange. Most are not trained in that, so that is an area we would ask for help in if the ophthalmologist would stand with us, come do some training, regular training if we had support from other partners to train the doctors in that, it would be beneficial and very welcome. Nurse_015

## 4 Discussion

In this study, most of the caregivers felt that the level of awareness about ROP among medical professionals and caregivers is low, which calls for designing interventions that could address this concern to improve the care and screening of preterm against ROP. Similar to findings from a cross‐sectional study conducted in the United States [16], caregivers in this study had limited knowledge about ROP. As highlighted by one of the paediatricians, the limited involvement of caregivers in the care of preterm at‐risk could have contributed to the knowledge gap. In addition, the paediatricians highlighted that the overwhelming number of preterm admitted amidst the low staffing levels of the NICU limits the interactions between the caregiver and health workers, subsequently affecting the delivery of health information about ROP to the caregiver. Another possible reason for the inadequate knowledge about ROP among caregivers could be the conflicting needs and high level of care [16, 17] required, which may affect the ability of caregivers to learn about ROP. Although most caregivers’ education level was above the secondary level, Uganda's low health literacy levels [18] might have significantly contributed to the low number of knowledgeable participants about ROP.

In this study, less than half of the health workers had substantial knowledge about ROP, which agrees with findings from India [18] and Palestine [19]. Unlike the studies conducted in India [18] and Palestine [19], which recruited only paediatricians, this study recruited paediatricians and neonatal nurses caring for preterm infants in the NICU. As expected, the percentage of paediatricians knowledgeable about ROP was higher than that of neonatal nurses due to the differences in training and curriculum. Despite that, knowledge about ROP among these health workers is low. The insufficient training of health workers on ROP, as noted in this study, where 24.6% had received training in ROP, could have led to this, thus negatively affecting the ability of health workers to identify and refer preterm infants at risk of ROP for screening. As noted in this study, the absence of hospital guidelines on ROP screening, management and prevention makes it difficult to disseminate and acquire knowledge about ROP. In addition, the lack of national policies and inadequate training on ROP could have contributed to the low number of paediatricians and neonatal nurses conducting fundus examinations to screen for ROP. However, this has not deterred them from referring caregivers to ophthalmologists and counselling caregivers about ROP, which aligns with the published guidelines on screening, managing and preventing ROP [11, 20].

Most health workers had a positive attitude towards preventing and treating ROP, probably due to the positive influence of the paediatricians and neonatal nurses who have undergone training. Furthermore, almost all the health workers indicated insufficient awareness about ROP among their colleagues and caregivers. Neonatal nurses are crucial in screening and preventing ROP and its complications [12, 13]. If appropriately trained, the task of screening, patient education and counselling could be shifted to them, as noted by one of the participants in the in‐depth interviews. The positive attitude and good practices of health workers in this study provide a platform for the target population to be willing to undertake training on ROP, which could reduce the incidence and development of blindness from ROP.

This study recruited participants from two national referral public hospitals with the largest NICU in the country, so the findings might be representative of health workers working in public facilities with a NICU. The study had limitations, such as using the same questionnaire for paediatricians and neonatal nurses and selection bias.

## 5 Conclusion

This study highlights insufficient knowledge about ROP in premature babies among caregivers and identifies potential barriers to enhancing ROP screening and care. The positive outcomes lay the groundwork for developing effective ROP management strategies and screening at‐risk premature infants. Government support for health worker training on ROP and distributing health education materials to caregivers is essential for early identification of at‐risk preterm infants. Future implementation‐based studies are anticipated to demonstrate the impact of strategies such as continuous medical education on ROP identification and management.

## Author Contributions

R.C.L. and I.A. contributed to the study's conception, design, data acquisition, analysis and interpretation, and manuscript drafting. I.A. contributed to the study's conception and design, data acquisition, data analysis and interpretation, and manuscript drafting. A.W.S. contributed to the design of the study, acquisition of data, interpretation of the data, and manuscript drafting. C.N., A.K., E.D. and M.N. contributed to the acquisition of data. J.M., C.B. and D.M. contributed to the analysis and interpretation of the data and the drafting of the manuscript. All authors have given final approval for the version to be published.

## Ethics Statement

Ethics approval was sought from the Mulago Hospital Research and Ethics Committee (MHREC‐2228) and the Uganda National Council of Science and Technology (HS2228ES) before data collection and administrative clearance were granted by the tertiary hospitals. Written informed consent was obtained before the initiation of study procedures, which followed the principles of the Declaration of Helsinki and Ugandan laws and regulations. The study team members were trained in Good Clinical Practice principles and Human Subject Protection.

## Conflicts of Interest

Geoffrey Wabulembo is an employee of Light for the World. The other authors declare no conflicts of interest.

## Data Availability

The data supporting this study's findings are available on request from the corresponding author. The data are not publicly available due to privacy or ethical restrictions.
